# The Effects of 3% Diquafosol Sodium Eye Drops on Tear Function and the Ocular Surface of Cu, Zn-Superoxide Dismutase-1 (*Sod1*) Knockout Mice Treated with Antiglaucoma Eye Medications

**DOI:** 10.3390/diagnostics10010020

**Published:** 2020-01-01

**Authors:** Yukari Yagi-Yaguchi, Takashi Kojima, Kazunari Higa, Murat Dogru, Osama MA. Ibrahim, Takahiko Shimizu, Kazuo Tsubota, Jun Shimazaki

**Affiliations:** 1 Department of Ophthalmology, Tokyo Dental College, Ichikawa general hospital, Ichikawa, Chiba 272-8513, Japan; yukarin4yaguchin@hotmail.com (Y.Y.-Y.); kazunari.higa@gmail.com (K.H.); meishano1@gmail.com (J.S.); 2 Department of Ophthalmology, Keio University School of Medicine, Shinjuku-ku, Tokyo 160-8582, Japan; kojima_takashi@keio.jp (T.K.); tsubota@z3.keio.jp (K.T.); 3 Department of Advanced Aging Medicine, Chiba University Graduate School of Medicine, Chiba 260-8670, Japan; shimizut@chiba-u.jp; 4 Cornea Center Eye Bank, Tokyo Dental College, Ichikawa general hospital, Ichikawa, Chiba 272-8513, Japan

**Keywords:** dry eye, anti-glaucoma eye drops, 3% diquafosol sodium eye drops, *Sod1*^−/−^ mice

## Abstract

Anti-glaucoma eye drop treatment often induces dry eyes and can lead to poor medication adherence. This study aimed to investigate the effects of 3% diquafosol sodium eye drops on tear function and the ocular surface epithelium in *Sod1*^−/−^ mice after treatment with anti-glaucoma eye drops. The mice were divided into four groups: group 1, control group; group 2, anti-glaucoma eye drop; group 3, anti-glaucoma eye drops followed by a secretagogue eye drop (3% diquafosol); and group 4, simultaneous anti-glaucoma and secretagogue eye drop. Mice underwent assessments of tear quantity, tear film breakup time, and vital staining score. Mice in groups 3 and 4 showed significantly better tear stability and lower corneal staining scores than mice in group 2 after eye drop instillations (*p* < 0.05). Mice in group 4 showed significantly better tear stability, lower corneal staining scores, and higher goblet cell densities than those in group 1 after eye drop instillations (p < 0.05). The conjunctival epithelium showed stratification and abundance of Muc5AC-positive goblet cells in group 4, whereas thinning with desquamation was observed with a few goblet cells in group 2. Thus, simultaneous administration of 3% diquafosol sodium eye drops with topical anti-glaucoma drops showed favorable effects on tear stability and the corneal epithelium against the ocular surface toxicity inflicted by the anti-glaucoma eye drops.

## 1. Introduction

Glaucoma is a chronic, progressive optic neuropathy. According to population-based surveys, it affects 60 million people around the world, with 8.4 million people experiencing bilateral blindness as a result [1]. Treatment for glaucoma includes the use of topical eye drops that enhance aqueous outflow, reduce aqueous production, or both. Long-term control of intraocular pressure with medication is necessary to protect the optic nerve head from further damage. However, many articles have reported toxic ocular adverse effects associated with various types of anti-glaucoma eye drops, especially due to their additives such as benzalkonium chloride (BAK) [2,3,4,5,6,7,8,9,10,11,12]. The resulting ocular surface disease in patients with glaucoma can lead to poor medication adherence [13]. Thus, management of ocular surface disease in glaucoma patients is important.

Tear film stability is important for maintaining a healthy corneal epithelium and the subepithelial nerves. Tear film stability is the primary factor underlying dry eye [14] and is related to many factors such as ocular surface wettability, tear viscosity, tear evaporation, and blinking. Secretory mucins stabilize the tear fluid with their water retention properties and prevent the ocular surface epithelium from friction due to blinking. Muc5AC is the most important secretory mucin secreted from the conjunctival goblet cells and is present in the aqueous layer of tears. Muc5AC secretion has been shown to decrease in dry eye disease [15,16].

Diquafosol is a uridine triphosphate–related compound that stimulates the P2Y2 receptors on the ocular surface (including the conjunctival epithelium, meibomian glands, and goblet cells) and contributes to conjunctival water transfer and mucin secretion [17,18]. Previous reports have concluded that 3% diquafosol sodium eye drops (DQS) were effective in the treatment of dry eye disease [19,20,21,22]. Furthermore, Shigeyasu et al. reported that DQS increased the tear meniscus volume and the secretion of sialic acid, which is a mucin-like substance, in the tears in healthy subjects after a single dose [23].

The superoxide dismutase (SOD) enzyme family, which consists of three isoenzymes (SOD1, SOD2, and SOD3), is one of the well-known antioxidant defense systems, and it plays critical roles in the removal of reactive oxygen metabolites. SOD1 is widely distributed in human cells and represents almost all of the SOD activity [24,25]. We previously reported that Cu, Zu-superoxide dismutase-1–knockout (*Sod1*^−/−^) mice showed ocular surface damage with a marked decrease in ocular surface Muc5AC expression and decreased tear secretion, and concluded that the *Sod1*^−/−^ mouse was a good model for studying age-related dry eye disease [26]. Considering the impact of antiglaucoma eye drops on tear function and the ocular surface in elderly patients, we conducted the current research using *Sod1*^−/−^ mice, an aging model, instead of using ordinary wild-type mice.

We also previously reported that DQS eye drop treatment improved tear function and the conjunctival epithelial status in *Sod1*^−/−^ mice that showed ocular surface disease due to age-related dry eyes [27].

In this study, we investigated the effect of DQS eye drops on the tear function and ocular surface alterations in *Sod1*^−/−^ mice treated with antiglaucoma eye drops containing BAK.

## 2. Results

### 2.1. Changes in Tear Film Breakup Time, Corneal Epithelial Staining Scores, and Tear Quantity

**Figure 1:**
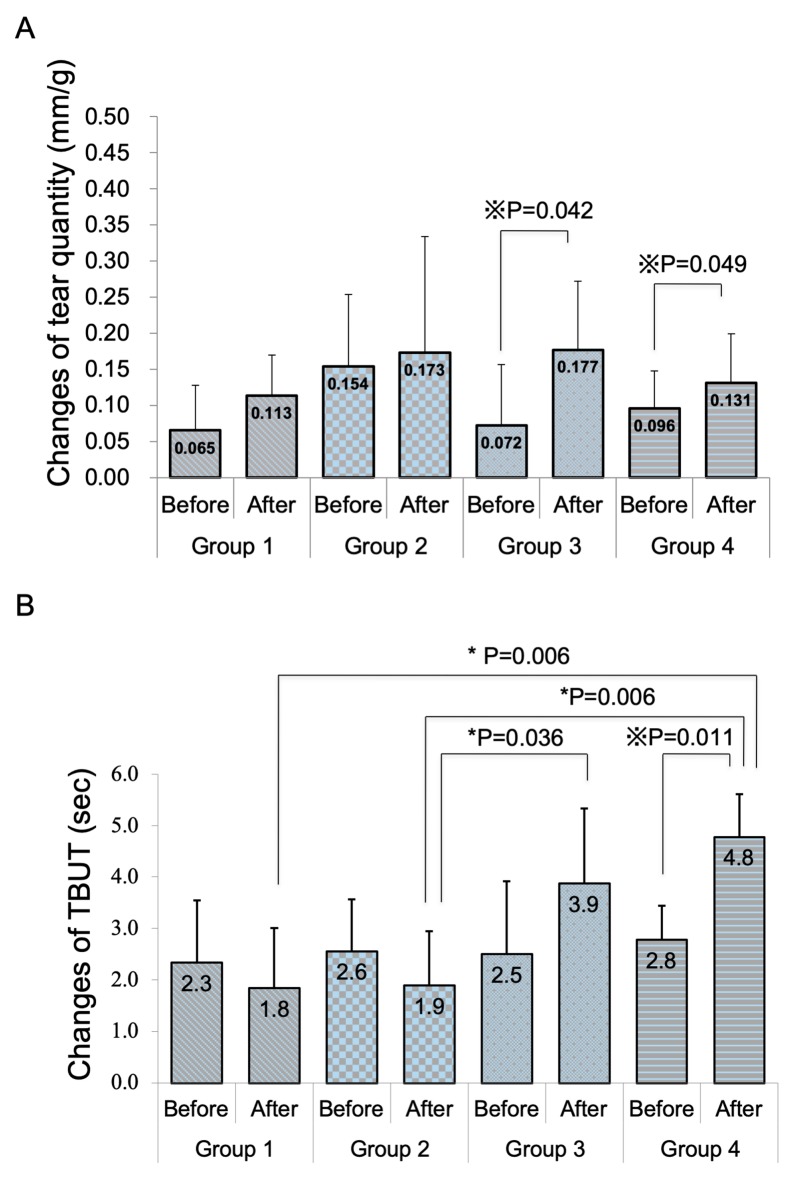
Changes in tear quantity (**A**) and tear film breakup time (TBUT) (**B**) in each group. There was a significant increase in the tear volume after eye drop instillation in groups 3 and 4. Note the significant improvement in tear stability with four weeks of simultaneous antiglaucoma and 3% diquafosol eye drop application in group 4. ※ Wilcoxon signed-rank test. * Mann–Whitney (adjusted with the Bonferroni method).

**Figure 2:**
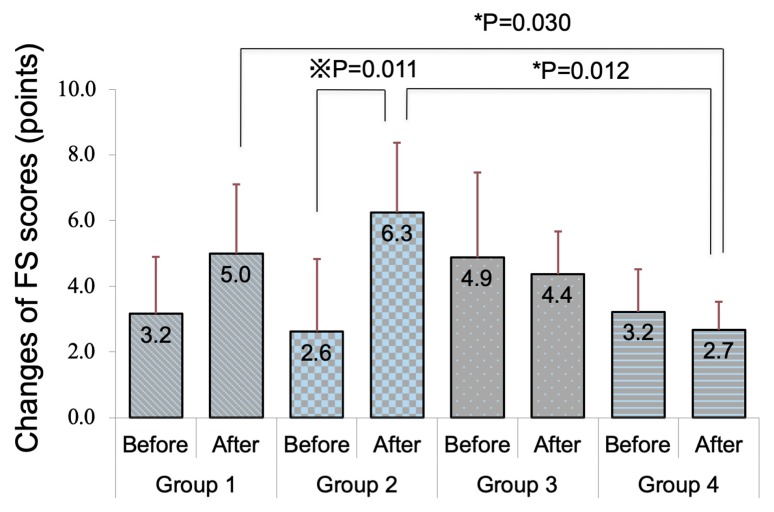
Changes in fluorescein scores in each group. Note the significant worsening in fluorescein staining (FS) score in group 2 after four weeks of antiglaucoma eye drop instillation. FS score showed no difference before and after eye drop application in groups 1, 3, and 4. FS score in group 4 was lesser than that in groups 1 and 2 after eye drop application. ※ Wilcoxon signed-rank test; * Mann–Whitney (adjusted with the Bonferroni method).

**Figure 3:**
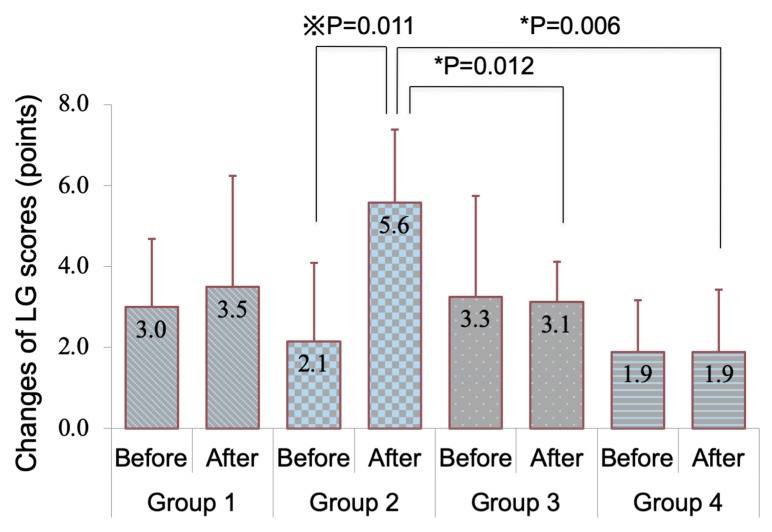
Changes in lissamine green (LG) scores in each group. Note the significant worsening in the LG staining score in group 2 after four weeks of antiglaucoma eye drop instillation. LG staining scores showed no differences in groups 1, 3, and 4. ※ Wilcoxon signs rank test; * Mann–Whitney (adjusted with the Bonferroni method).

The weights of mice in groups 2, 3, and 4 were significantly reduced after a month (*p* = 0.007, 0.034, and 0.007, respectively). Figure 1A shows the changes in weight-adjusted tear volume. Mice in groups 3 and 4 showed significant increases in tear quantity between before and after eye drop instillation (Figure 1A, *p* = 0.042 and 0.049, respectively). There was no significant difference in tear volume between any of the groups. Only mice in group 4 showed a significant improvement in tear stability after eye drop instillation in comparison with pre-instillation values (Figure 1B, *p* = 0.011). Mice in groups 3 and 4 showed significantly better tear stability than mice in group 2 after eye drop instillations (*p* = 0.034 and 0.006, respectively). Mice in group 4 also showed significantly better tear stability than mice in group 1 after eye drop instillation (*p* = 0.006). Figure 2 and Figure 3 show the changes in corneal vital staining scores. Only mice in group 2 showed significant worsening of FS and LG scores between before and after eye drop instillation (*p* = 0.011 and 0.011, respectively). Mice in group 4 showed significantly lesser FS scores than mice in groups 1 and 2 after eye drop instillation (*p* = 0.030 and 0.012, respectively). Mice in groups 3 and 4 also showed significantly lower LG scores than mice in group 2 after eye drop instillation (*p* = 0.012 and 0.006, respectively).

### 2.2. Conjunctival Histopathological Alterations

**Figure 4:**
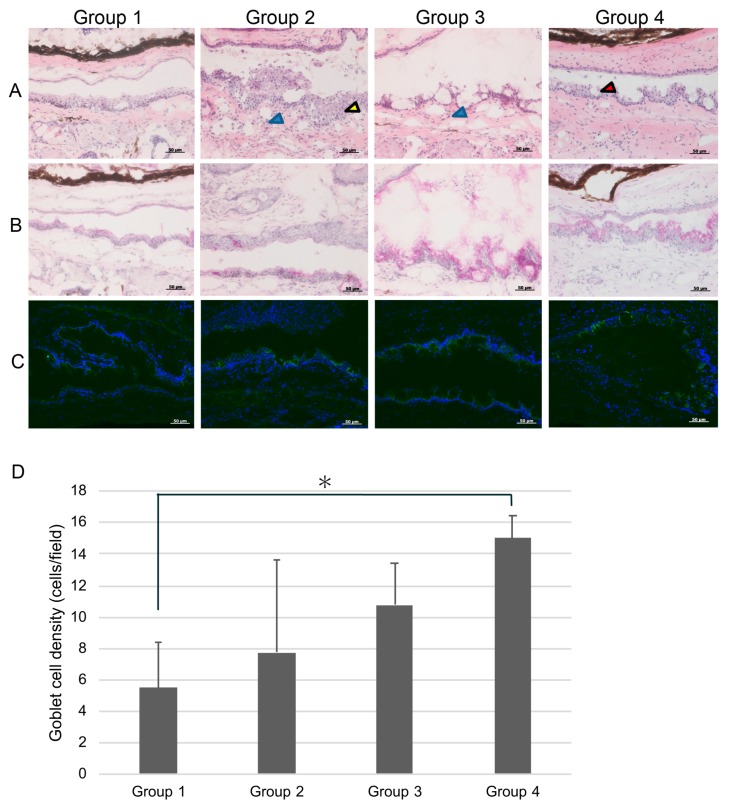
Comparison of conjunctival histopathological alterations. (**A**) Conjunctival hematoxylin-eosin staining after eye drop instillation. Note the age-related thinning of the conjunctival epithelium and the marked decrease in goblet cells in group 1. Note the thickening of the conjunctival epithelium (yellow arrow) in group 2, and the increased desquamation of epithelial cells and inflammatory cells in the stromal tissue (blue arrow) in groups 2 and 3. Note the marked increase in goblet cells (red arrow) in group 4. (**B**) Conjunctival periodic acid Schiff (PAS) staining after eye drop instillation. Note the scarcity of goblet cells in groups 1 and 2. Note the abundance of PAS staining-positive goblet cells in groups 3 and 4. (**C**) Conjunctival immunohistochemical staining for Muc5AC. Note the scarcity of Muc5AC (+) staining in the conjunctival epithelium in groups 1 and 2. Note the dense staining for Muc5AC in the conjunctival epithelium in groups 3 and 4. (**D**) The goblet cell density in group 4 was significantly higher than that in group 1. * represents *p* < 0.05 with Mann–Whitney test.

Figure 4A shows conjunctival HE staining after eye drop instillation. Conjunctival specimens of mice in group 2 showed thinning of the epithelium with increased desquamation and infiltration with inflammatory cells in the stromal tissue. Group 4 showed stratification of the conjunctival epithelium and abundance of goblet cells. Figure 4B showed conjunctival PAS staining after eye drop instillation. Although conjunctival specimens in the mice belonging to groups 1 and 2 showed a lack of goblet cells, those in groups 3 and 4 showed a notable increase in goblet cells. Figure 4C shows abundant conjunctival goblet cell staining with Muc5AC in groups 3 and 4. Figure 4D shows a comparison of goblet cell density (GCD). The GCD in group 4 was significantly higher than that in group 1 (*p* = 0.0135).

## 3. Discussion

In this study, we reported the efficacy of DQS ophthalmic solution on tear function and ocular surface disorders induced by antiglaucoma eye drops in an aging mouse model.

Glaucoma is the second leading cause of blindness in the world [13]. The mainstay of initial treatment consists of topical antiglaucoma eye drops, mainly prostaglandin inhibitors. According to a review article, 49% of patients with ocular hypertension require at least two antiglaucoma eye medications within five years of diagnosis [13]. Prostaglandin and prostaglandin-containing fixed combination therapy accounted for 51.8% of all anti-glaucoma agents in 2013 in Japan [28].

Antiglaucoma eye drops have been reported to be associated with corneal epithelial damage and a significant reduction in conjunctival goblet cells [29]. Preservatives in antiglaucoma eye drops have been reported to affect tear turnover and stability, and result in the infiltration of inflammatory cells and fibroblasts in the conjunctiva [30].

The ocular surface epithelial damage has been reported to be remarkable, especially in patients using more than two antiglaucoma eye drops. The ocular surface disease is more pronounced in the presence of dry eyes and in elderly patients, and it can lead not only to poor medication compliance, but also to a higher rate of failure in subconjunctival glaucoma surgery [30,31,32].

Current treatment modalities for the treatment of ocular surface epithelial damage in patients suffering from such complications of anti-glaucoma eye drops consist of the instillation of non-preserved artificial tear drops, hyaluronic acid eye drops, or water and/or mucin secretagogues like 3% DQS or 2% rebamipide eye drops.

In this study, we aimed to investigate the efficacy of DQS on the ocular surface disease induced by topical glaucoma eye drops. Although no changes in TBUT were found in group 2, a significant deterioration in the vital staining score that was not observed in group 1 was found in group 2, which suggests that dry eye was worsened by antiglaucoma eye drop instillation. In our experience, DQS eye drops administered at the onset or initiation of antiglaucoma medication provided a significant improvement in tear stability and a remarkable decrease in FS scores compared to mice receiving antiglaucoma eye drops alone. This observation was also consistent with the findings of LG staining, which reflects the breakdown of the ocular surface barrier and the loss of mucins on the ocular surface epithelium.

The higher LG scores in the solitary anti-glaucoma eye drop group compared to those in groups 3 and 4, in which the mice received DQS eye drops, indicated that DQS eye drops might have had a favorable influence on mucins released by the ocular surface epithelium. We mainly focused on the most abundant mucin in the ocular surface and tears, namely Muc5AC. Indeed, PAS and IHC staining showed abundant and dense staining with numerous positively stained goblet cells and significantly higher numbers of goblet cells in group 4 treated with DQS eye drops.

TBUT improved significantly in group 4, but not in group 3. Since 3% diquafosol was used for two weeks in group 3 and four weeks in group 4, the duration of the instillation of 3% diquafosol also might have affected the results.

Goblet cell density deteriorated after the instillation of antiglaucoma eye drops in group 2, but no significant difference was observed before and after instillation in groups 3 and 4. This indicates that 3% diquafosol prevented the decrease in goblet cell density. However, there was no difference in the goblet cell density after four weeks among groups 2, 3, and 4, probably because of the wide individual variations in goblet cell numbers.

A limitation of this study was that we were unable to evaluate tear function and ocular surface damage at two weeks after antiglaucoma instillation. Understanding the time-course of changes in ocular surface damage and tear function may be important for evaluating the usefulness of 3% DQS ophthalmic solutions in patients using antiglaucoma eye drops, and future studies should implement a more detailed evaluation of tear function and ocular surface abnormalities at various time points.

The initial observations from the current study may justify the implementation of a similar treatment protocol in human clinical trials comparing DQS eye drop instillations with different timings to the use of non-preserved antiglaucoma eye drops or non-preserved artificial tears alone or in combination. Such future studies will enrich our knowledge of the treatment of ocular surface disease associated with anti-glaucoma eye drops.

## 4. Materials and Methods

### 4.1. Animals

Thirty-two eyes of nineteen 40-week-old *Sod1*^−/−^ female mice with a C57BL/6 background were examined. The *Sod1*^−/−^ mice were received from the department of Advanced Aging Medicine, Chiba University Graduate School of Medicine (Chiba, Japan). No abnormality was found in the anterior segment when examined with the slit-lamp microscope. Mice were housed in the same standard environmental conditions throughout the study. The temperature and humidity at the animal facility were maintained at 25 °C and 40%, respectively. All procedures were performed in accordance with the Association for Research in Vision and Ophthalmology Statement for the Use of Animals in Ophthalmic and Vision Research.

In this study, preservative-free 3% diquafosol ophthalmic solution (Santen, Osaka, Japan) was used to treat dry eye and the ocular surface disease induced by antiglaucoma eye drops, namely, 0.005% latanoprost (Pfizer, New York City, NY, USA) 0.5% timolol (Santen), or 1% dorzolamide (Santen). Antiglaucoma eye drops contained BAK at the following concentrations: latanoprost, 0.002%; timolol, 0.001%; and dorzolamide, 0.001%. The eyes of mice were equally divided into four groups and underwent instillations of three types of antiglaucoma eye drops (latanoprost 0.005%, once a day; timolol 0.5%, twice a day; or dorzolamide 1%, thrice a day) and DQS four times a day. When using two or more types of eye drops, the eye drops were instilled at 10-min intervals.

**Figure 5:**
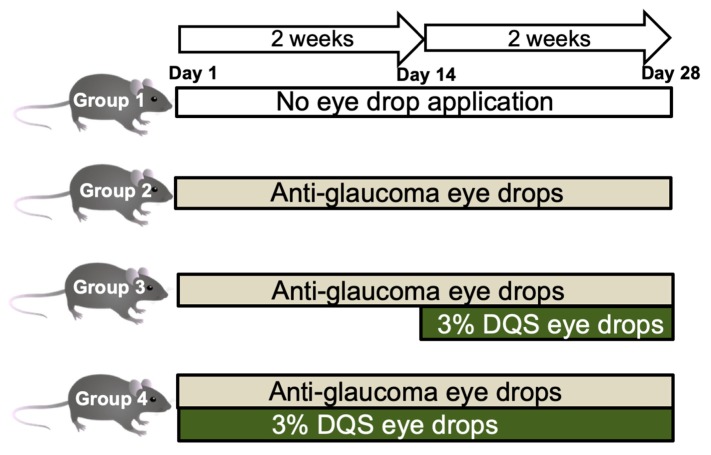
The schema of the study protocol.

Group 1 was the control group that did not receive the eye drop application; animals in group 2 received antiglaucoma eye drops for four weeks; those in group 3 received anti-glaucoma eye drops for two weeks with subsequent combined use of 3% DQS with antiglaucoma eye drops for another two weeks; and those in group 4 received simultaneous application of anti-glaucoma and DQS eye drops for four weeks. All mice also underwent body weight measurements. The scheme of the study protocol is shown in Figure 5.

The mice underwent instillations of the three types of anti-glaucoma eye drops (latanoprost 0.005%, once a day; timolol 0.5%, twice a day; or dorzolamide 1%, thrice a day), and 3% DQS as follows:

Group 1: Control group without eye drop application.

Group 2: Antiglaucoma eye drops * for four weeks

Group 3: Two weeks of antiglaucoma eye drops followed by DQS eye drops six times a day for two weeks.

Group 4: Simultaneous application of antiglaucoma and DQS eye drops for four weeks.

All studies were performed in accordance with the Association for Research in Vision and Ophthalmology (ARVO) Statement for the Use of Animals in Ophthalmic and Vision Research. The Animal Experimentation Ethics Committee of the Tokyo Dental College approved the current research procedures (287606, 1 April 2017).

### 4.2. Tear Film Breakup Time, Corneal Epithelial Damage Assessment, and Tear Volume Measurements

Corneal fluorescein staining was performed with 2 μL of 0.5% sodium fluorescein staining solution. The eyes were observed under cobalt blue light by slit-lamp biomicroscopy after fluorescein instillation. The tear film breakup time after a natural blink was recorded. Next, 2 μL of 1 % sodium lissamine green staining solution was instilled on the ocular surface by a micropipette after fluorescein staining. The fluorescein and lissamine green staining scores were analyzed using a grading system of 0–3 points for the superior, central, and inferior corneal areas. The scores ranged from a minimum of 0 to a maximum of 9 points.

Phenol red-impregnated cotton threads (Zone-Quick, Showa Yakuhin Kako Co. Ltd., Tokyo, Japan) were used to measure tear volume without anesthesia on days 0 and 28 after instillations. The threads were immersed into the tear meniscus in the lateral canthus for 60 s, and the tear-soaked thread lengths were measured in millimeters. For statistical analyses, weight-adjusted tear volume was calculated.

### 4.3. Conjunctival Specimen and Eyeball Collections

Mice were anesthetized intraperitoneally and were sacrificed using 50 mg/mL of pentobarbital (Kyoritsu Seiyaku Co., Tokyo, Japan) at 44 weeks after four weeks of eye drop instillations. The whole globes were rapidly removed after sacrifice. For histopathologic assessment, globes were fixed in Tissue-Tek OCT compound (Sakura Finetec, Tokyo, Japan), frozen in liquid nitrogen, and then stored in a −80 °C freezer.

### 4.4. Histopathological Assessment of Specimens and Calculation of Goblet Cell Densities

Five-micrometer sections were excised with a cryostat. To evaluate the goblet cells, hematoxylin and eosin (HE) and periodic acid Schiff (PAS) staining were performed. For PAS staining, these sections were processed according to conventional histological techniques. Briefly, after fixation with 10N formalin, slides were immersed in 5% periodic acid for 5 min, rinsed in three changes of distilled water, and immersed in Schiff solution for 15 min. The slides were rinsed with distilled water and underwent hematoxylin staining for 1 min.

Five randomly selected nonoverlapping areas of in each section in 420 × 332-μm frames were digitally photographed (Axioplan 2 Imaging; Carl Zeiss, Jena, Germany). The goblet cell densities were then calculated using ImageJ software (National Institute of Health, Bethesda, MD, USA, Version 1.51).

### 4.5. Fluorescent Immunohistochemistry (IHC) Staining for Muc5AC

To evaluate the localization and expression level of Muc5AC in conjunctival tissues of *Sod1*^−/−^ mice, Muc5AC immunohistochemical staining was also performed. Frozen sections (thickness, 5 μm) were fixed with 10N formalin (Wako, Osaka, Japan) for 10 min. After blocking with 10% normal donkey serum (Merck KGaA, Darmatadt, Germany) and 1% bovine serum albumin (Sigma, St Louis, MO, USA) at room temperature (RT) for 60 min, sections were incubated with anti-Muc5AC IgG (1:30, SantaCruz, San Diego, CA) at RT for 90 min at RT. After twice washing with phosphate-buffered saline (PBS) for 5 min, sections were incubated with fluorescein isothiocyanate-conjugated secondary antibody (1:100, Jackson Immuno Research Laboratories, West Grove, PA). After three additional washes with PBS for 5 min, the sections were incubated with 0.5 µg/mL 4′, 6-diamidino-2-phenylindole (DAPI; Dojindo Laboratories, Tokyo, Japan) at RT for 5 min, then rinsed with PBS for 5 min three times, and finally coverslipped with a mounting medium containing an anti-fading agent (Fluoromount/Plus, Diagnostic Biosystems, Pleasanton, CA, USA). Prepared sections were digitally photographed with a florescence microscope (Axioplan2 imaging, Carl Zeiss Inc., Thornwood, NY, USA).

### 4.6. Statistical Analysis

For the statistical analysis, SPSS version 24 statistical software (IBM Japan, Ltd., Tokyo, Japan) was used. The Wilcoxon matched pairs test and Mann–Whitney’s U test adjusted with the Bonferroni method were used for analyses of nonparametric values. *p* values less than 5% were considered to be statistically significant.

## 5. Conclusions

The current study showed better tear stability, vital staining scores, and higher goblet cell densities when 3% DQS eye drops were instilled from the time of initiation of the anti-glaucoma eye drop treatment, which suggests favorable effects on ocular surface disease resulting from anti-glaucoma eye medications
